# Cumulative exposure to AHA Life's Essential 8 is associated with nonalcoholic fatty liver disease: a large cohort study

**DOI:** 10.1186/s12986-024-00821-z

**Published:** 2024-06-27

**Authors:** Wang Yaqin, Deng Shuwen, Yuan Ting, Zhu Xiaoling, Deng Yuling, Liu Lei, Wang Changfa

**Affiliations:** 1 Health Management Center, The Third Xiangya Hospital, Central South University, Yuelu District, No.138 Tongzipo Road, Changsha, 410013 Hunan China; 2 General Surgery Department, The Third Xiangya Hospital, Central South University, Yuelu District, No.138 Tongzipo Road, Changsha, 410013 Hunan China

**Keywords:** AHA Life's essential 8, Nonalcoholic Fatty Liver Disease, Development, Regression, Cohort

## Abstract

**Background and Aim:**

We aimed to explore the associations of baseline and cumulative cardiovascular health with nonalcoholic fatty liver disease (NAFLD) development and regression using the new Life’s Essential 8 score.

**Methods:**

From a health screening database, participants who underwent at least 4 health examinations between 2012 and 2022 were recruited and categorized into two cohorts: (a) the NAFLD development cohort with no history of NAFLD prior to Exam 4 and (b) the NAFLD regression cohort with diagnosed NAFLD prior to Exam 4. The LE8 score was calculated from each component. The outcomes were defined as newly incident NAFLD or regression of existing NAFLD from Exam 4 to the end of follow-up.

**Results:**

In the NAFLD development cohort, of 21,844 participants, 3,510 experienced incident NAFLD over a median follow-up of 2.3 years. Compared with the lowest quartile of cumulative LE8, individuals in the highest quartile conferred statistically significant 76% lower odds (hazard ratio [HR] 0.24, 95% confidence interval [CI], 0.21–0.28) of NAFLD incidence, and corresponding values for baseline LE8 were 42% (HR 0.58, 95% CI 0.53–0.65). In the NAFLD regression cohort, of 6,566 participants, 469 experienced NAFLD regression over a median follow-up of 2.4 years. Subjects with the highest quartile of cumulative LE8 had 2.03-fold (95% CI, 1.51–2.74) higher odds of NAFLD regression, and corresponding values for baseline LE8 were 1.61-fold (95% CI, 1.24–2.10).

**Conclusion:**

Cumulative ideal cardiovascular health exposure is associated with reduced NAFLD development and increased NAFLD regression. Improving and preserving health behaviors and factors should be emphasized as an important part of NAFLD prevention and intervention strategies.

**Graphical Abstract:**

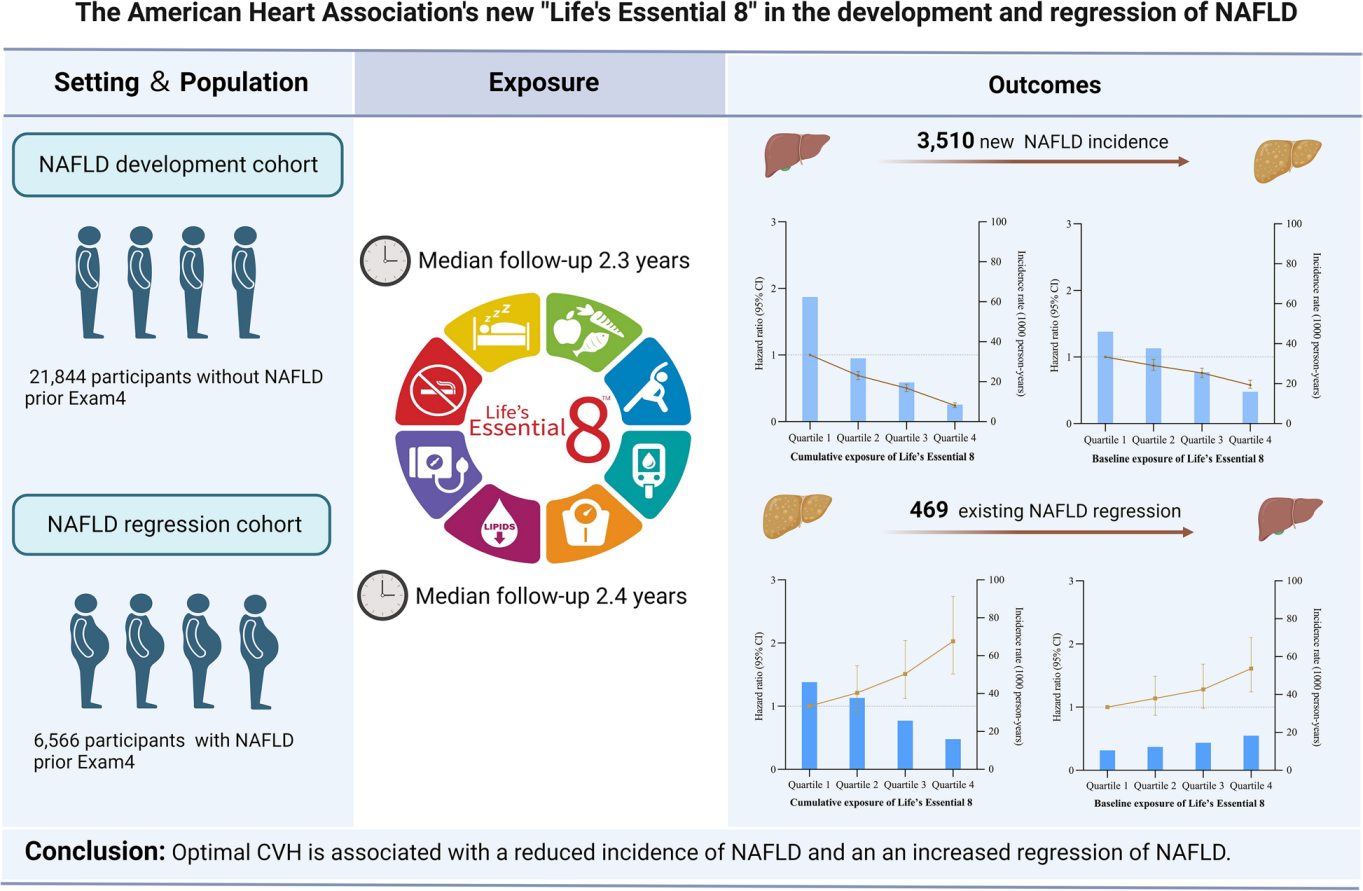

**Supplementary Information:**

The online version contains supplementary material available at 10.1186/s12986-024-00821-z.

## Introduction

In 2010, the American Heart Association (AHA) formulated a definition of cardiovascular health (CVH) called Life’s Simple 7 (LS7) based on 7 risk factors that can be improved or amended via lifestyle interventions [1]. The 7 components covered healthy diet, participation in physical activity, avoidance of nicotine, healthy weight, and healthy levels of blood lipids, blood glucose, and blood pressure. Over the past 13 years, LS7 has been proven to be a paramount tool for the health care system, researchers and policymakers to focus efforts on how to perform primordial prevention and monitor CVH in individuals and populations while also showing protective effects against cerebrovascular disease (CVD), as well as cancer, end-stage renal disease, dementia, chronic obstructive pulmonary disease and other chronic diseases. In 2022, AHA introduced a novel and enhanced construct to assess CVH called Life’s Essential 8 (LE8) as an update of LS7, which added sleep quality as a new component and redefined the scoring algorithm [2]. The new modified concept of LE8 will carry on the catalytic role of positive health promotion across the life span.

The prevalence of NAFLD in the general population is approximately 25% and is soaring at an unanticipated rate in China from 18 to 29% within a decade [3]. Numerous studies have revealed that NAFLD is not generally considered ‘benign’ and has substantial long-term non-liver (CVD, extrahepatic cancers) and liver-related (cirrhosis, hepatocellular carcinoma) comorbidities. Therefore, NAFLD confers a global disease burden, and prevention and intervention actions should be addressed to reverse this ‘pandemic’ in the future.

Since NAFLD shares similar lifestyle and cardiovascular metabolic risk factors with CVD, prior studies have found an association between ideal CVH and NAFLD, but there are still several limitations. These clinical studies were limited by small sample sizes, retrospective studies or cross-sectional analyses, and the outcome mainly focused on the risk of NAFLD development, not regression [4–6]. Moreover, the CVH metrics were only measured at a single time point, the potential intraindividual changes over time in CVH status have not been examined comprehensively, and such variability may contribute to biased estimates of the association [7–9]. In view of the aforementioned gaps, we aimed to conduct a comprehensive evaluation of both baseline and cumulative CVH exposure based on the new definition of LE8 in relation to the incidence and regression of NAFLD within a large physical examination cohort of the Chinese population.

## Methods

### Study Population

We used the data from an ongoing longitudinal study in Hunan, China, of which a detailed description has been published [10]. This study cohort consisted of repeated routine health check-up examinations at the Health Management Center in the Third Xiangya Hospital of Central South University (Changsha, China), the largest medical institution in central China. The electronic health records database is dynamic and includes sociodemographics, lifestyle factors, prescriptions, diagnoses from specialist referrals, hospital admissions, anthropometric characteristics and laboratory test results.

**Fig. 1:**
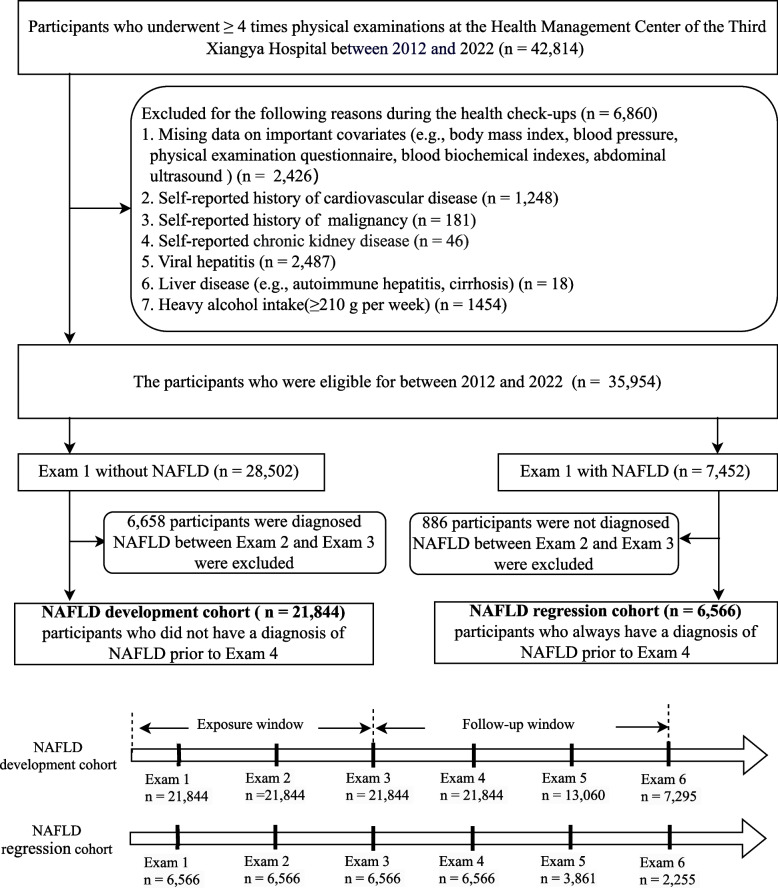
Flow Chart of Our Study

From January 2012 to December 2022, a total of 21,844 participants were identified in the NAFLD development cohort as having at least 4 times physical examinations, not having a diagnosis of NAFLD prior to Exam 4, and having complete follow-up data available; similarly, a total of 6,566 participants were identified in the NAFLD regression cohort who always had a diagnosis of NAFLD prior to Exam 4 (Fig. 1). The study was approved by the Ethics Committees of the Third Xiangya Hospital of Central South University (no. R18030) following the guidelines outlined by the Helsinki Declaration. Each study participants agreed to participate in this study and provided written informed consent.

### Covariates

A standard national physical examination questionnaire, anthropometric and biochemistry data and abdomen ultrasound were collected at baseline and during each of the following exams. The questionnaire covered demographic (age, sex and education), lifestyle (dietary intake, physical activity, smoking status, sleeping time and alcohol consumption), and clinical characteristics (previous diseases, the use of antihypertensive, cholesterol-lowering, and glucose-lowering medications) as previously reported [11].

Trained examiners measured participants’ height, weight, waist circumference (WC) and blood pressure. Body mass index (BMI) was obtained by dividing the weight (kg) by the square of height (m^2^). WC was measured from the bottom edge of the last rib and iliac crest. Blood pressure (BP) was measured on the right upper arm in the sitting position after 10–15 min of rest using a validated digital automatic analyzer (Omron 9020).

Fasting blood samples were collected and immediately sent to the central laboratory of the Third Xiangya Hospital for the determination of blood lipids, plasma glucose, alanine aminotransferase (ALT) and serum creatinine with the use of a Hitachi autoanalyzer (Hitachi 747; Hitachi, Tokyo, Japan). Detailed information on the sample analysis is provided in Supplementary Item 1. Non-high-density lipoprotein (non-HDL-C) was calculated as total cholesterol (TC) minus high-density lipoprotein cholesterol (HDL-C) [12]. The estimated glomerular filtration rate (eGFR) was used as an index of renal disease based on the Modification of Diet in Renal Disease formula for Chinese subjects: eGFR = 175 × Scr^−1.234^ × age^−0.179^ [if female, × 0.79] [13]. Details of other covariate definitions are presented in Supplementary Item 2.

### *AHA* Life's Essential 8 Exposure Assessment

AHA Life's Essential 8 was assessed based on 4 health behaviors (diet, physical activity, nicotine exposure and sleep duration) and 4 health factors (BMI, non-HDL-C, blood glucose and blood pressure) [2]. The scoring of each metric ranged from 0 to 100 points according to NHANES data (Supplementary Table 1), and the composite LE8 score was calculated as the unweighted average of all 8 score components, which also varied from 0 to 100 points. Similarly, the composite health behavior was computed as the unweighted average of the 4 health behaviors and the composite health factor as the 4 health factors.

The diet metric was assessed by Dietary Approaches to Stop Hypertension (DASH), which consists of 8 components (including vegetables, total fruit, nut and legumes, whole grains, dairy, red and processed meat, sugar-sweetened beverage and fruit juice, and sodium) scaled from 8 to 40 points (Supplementary Table 2) [14]. Higher scores denoted better dietary quality and higher adherence to healthy dietary patterns.

We defined the LE8 score at each examination (i.e., LE8_Exam1_, LE8_Exam2_ and so on). To account for the potential cumulative effect of the dynamic changes, we derived a time-weighted cumulative exposure of the LE8 score (cum-LE8) for each participant during the entire follow-up period. The cum-LE8 was defined as (LE8_Exam1_ × time_1-2_ + LE8_Exam2_ × time_2_-_3_ + LE8_Exam3_ × time_3-4_ + LE8_Exam4_ × time_4-5_ + LE8_Exam5_ × time_5-6_)/the follow-up duration (time_1-2_ + time_2-3_ + time_3-4_ + time_4-5_ + time_5-6_), where time_n-n+1_ indicates the interval time between the two consecutive exams from Exam_n_ to Exam_n+1_ in years. The same scoring algorithm was calculated for cum-health behaviors and cum-health factors.

### Determination of NAFLD and outcome

Hepatic steatosis was assessed by liver fat attenuation measured on abdominal ultrasonography as previously described [15]. In brief, ultrasonography was conducted by experienced radiologists who were blinded to the study data using a high-resolution B-mode tomographic ultrasound system with a 3.5-MHz probe (Logiq 9, GE Medical System, Milwaukee, WI, USA). Positive hepatic steatosis was determined as increased hepatic echogenicity (‘bright liver’) relative to the presence of two of the following three criteria: liver-to-kidney contrast, vascular blurring and deep beam attenuation based on the Asia–Pacific Working Party recommendations [16, 17]. NAFLD was diagnosed as the presence of fatty liver hepatic steatosis without excessive drinking (≥ 30 g/day in men, ≥ 20 g/day in women) or concomitant liver diseases (viral hepatitis, drug-induced hepatitis, autoimmune hepatitis and hepatolenticular degeneration, etc.) [18]. NAFLD status were evaluated in both exposure window and follow-up window. The new development of NAFLD was defined as those without NAFLD during exposure window but with new incident NAFLD in follow-up window. The regression of NAFLD was defined as those with NAFLD during exposure window but without NAFLD in follow-up window.

The outcome was the new development of NAFLD in the NAFLD development cohort and the new regression of NAFLD in the NAFLD regression cohort from exam 4 to the last exam. During the follow-up period, the first occurrence and the first regression of existing NAFLD were included for each event analyses. Follow-up time was calculated as the interval from the date of the baseline assessment (Exam 1) to the first occurrence of NAFLD or the last exam if incident NAFLD had not been identified for the NAFLD development cohort; on the opposite, to the first regression of NAFLD or the last exam if regressed NAFLD had not been identified for the NAFLD regression cohort.

### Statistical analyses

Baseline characteristics are presented as the means (standard deviations) or medians (interquartile ranges) for continuous variables and frequencies (proportions) for categorical variables. Participants were categorized into quartiles of cum-LE8 point score in the NAFLD development and regression cohorts. These quartile subgroups were compared by ANOVA or the Kruskal‒Wallis test or χ2 test. The incidence rates (IR) of the study outcomes were calculated as the total number of events per 100,000 person-years of the follow-up.

Cox proportional hazards models were used to estimate the independent effect of exposure to ideal cardiovascular health and risk of (a) incident NAFLD and (b) regressive NAFLD, including cum-LE8 and baseline-LE8. The hazard ratios (HRs) and 95% CI were calculated with exposure as a categorical variable (in quartiles) and a continuous variable (per 1 score). Proportional hazards (PH) assumptions were examined by Schoenfeld residuals and graphical inspection of log-minus-log plots. Covariables were selected a priori based on possible risk factors for NAFLD or associated with CVH in univariate analysis with a value of *p* < 0.10. We examined the association in 3 steps: model 1, adjusted for age and sex at baseline; model 2, additionally adjusted for education level and current drinking; and model 3, further adjusted for WC, eGFR, ALT at Exam 1, and antidiabetic, lipid-lowering, or antihypertensive medication usage before the last exam. In addition, we performed subgroup analyses to identify interactions with sex (female vs. male) and age (< 40 vs. ≥ 40 years based on mean age in the study) on the risk of incident NAFLD and regressive NAFLD. The multiplicative interactions were assessed by adding interaction terms into the Cox models. Tests for trend were based on variables containing the median value for each quartile as continuous variables in the Cox regression models.

Statistical analyses were performed using R software (version 3.6.3). All analyses were two-tailed, and the significance difference was set at *P* < 0.05.

### Sensitivity analyses

Several sensitivity analyses were conducted to test the robustness of our findings. First, the effects of both cum-health behaviors and cum-health factors on the risk of the study outcomes were examined separately. Second, the different categories of the baseline and cum-LE8 scores were reclassified as 0–49 (low), 50–74 (intermediate), and 75–100 points (high) according to the American Heart Association’s recommendations [2]. Third, to check whether the exclusion of subjects who were diagnosed with new incident NAFLD (*n* = 6,658) in the NAFLD development cohort or diagnosed with regressive NAFLD (*n* = 886) in the NAFLD regression cohort prior to Exam 4 influenced the main results, we reconducted analysis of the relationship between baseline LE8 at Exam 1 and (a) incidence of NAFLD and (b) regression of existing NAFLD at Exam 2.

## Results

### Baseline characteristics

There were 21,844 participants in the NAFLD development cohort, the mean age was 39.4 years (standard deviation, SD ± 12.6), and 58.8% of the participants were female; of the 6,566 subjects included in the regression cohort, the mean age was 43.9 years (SD ± 11.5), and 13.2% were women. Table 1 summarizes the baseline participant characteristics of the two cohorts. The NAFLD development group had a better metabolic health profile and higher baseline LE8 score and cum-LE8 score than the NAFLD regression cohort.

**Table 1:** Baseline characteristics of NAFLD development and regression cohort

Characteristics	NAFLD development cohort	NAFLD regression cohort	*P*-Value
	***n* = 21, 844**	***n* = 6, 566**	
Age, mean (SD), y	39.4 (12.6)	43.9 (11.5)	< 0.001
Female, n (%)	12,840 (58.8)	864 (13.2)	< 0.001
University degree, n (%)	18,844 (86.3)	5615 (85.5)	0.124
BMI, kg/m^2^	21.9 (2.4)	26.7 (2.6)	< 0.001
WC, cm	75.1 (7.8)	90.1 (7.2)	< 0.001
Systolic blood pressure, mm Hg	116.0 (14.1)	126.9 (13.0)	< 0.001
Diastolic blood pressure, mm Hg	71.2 (9.7)	79.9 (9.9)	< 0.001
Fasting glucose, mmol/L	5.1 (0.7)	5.5 (1.1)	< 0.001
Non- HDL cholesterol, mmol/L	3.0 (0.9)	3.8 (0.9)	< 0.001
eGFR, mL/min/1.73m^2^	121.1 (104.2– 141.5)	108.2 (95.2– 123.9)	< 0.001
ALT, U/L	17.0 (13.0– 23.0)	34.0 (24.0– 50.0)	< 0.001
Hypertension, n (%)	2005 (9.2)	1612 (24.6)	< 0.001
Diabetes mellitus, n (%)	463 (2.1)	434 (6.6)	< 0.001
Dyslipidemia, n (%)	2007 (10.1)	3091 (47.1)	< 0.001
Follow-up period (years)	2.3 (1.2– 3.3)	2.4 (1.2– 3.2)	0.058
Times of screening exams	5.2 (1.3)	5.2 (1.4)	0.279
Baseline LE8 score	77.5 (67.5– 83.1)	58.8 (51.9– 66.3)	< 0.001
Cum-LE8 score	76.0 (69.6– 81.4)	60.4 (53.4– 67.2)	< 0.001

Values are n (%), mean ± SD, or median (first quartile, third quartile)

*Abbreviations*: *BMI* Body mass index, *WC* waist circumference, *HDL* high-density lipoprotein, *ALT* alanine aminotransferase, *eGFR* estimated glomerular filtration rate

In addition, baseline participant characteristics of the NAFLD development cohort stratified by quartile of cum-LE8 exposure are shown in Supplementary Table 3. In general, participants in the highest quartile of cum-LE8 exposure tend to be younger, predominantly female, more educated and have a lower prevalence of traditional vascular risk factors. Similarly, the distribution of participant characteristics in the NAFLD regression cohort showed statistically significant differences across cum-LE8 score quartiles as shown in Supplementary Table 4.

### Association Between LE8 Exposure and NAFLD Development

Table 2 presents the adjusted HRs of incident NAFLD with quartiles of cum-LE8 exposure. During a median follow-up of 2.3 (IQR, 1.2–3.3) years, 3,510 (63.1 per 1,000 person-years) subjects experienced a NAFLD incidence from Exam 4 to the last exam. After multivariable adjustment, the HRs of incident NAFLD decreased steadily as cum-LE8 exposure increased. Quartiles 2, 3, and 4 were significantly associated with a decreased risk for NAFLD (31%, 50% and 76%, respectively) compared with Quartile 1 (the lowest quartile). For every 1 score increase in cum-LE8, the risk of NAFLD decreased by 4% (HR 0.96, 95% CI 0.95–0.97) in a dose‒response relationship (*P* for trend < 0.001). For subgroup analysis, similar associations were observed across sex and age subgroups; however, there were no significant interactions for sex and age with the impact of cumulative LE8 exposure on the risk of incident NAFLD.

**Table 2:** Risks of NAFLD Development according to the cumulative exposure of LE8 (*n* = 21,844)

	Groups of cum-LE8 exposure				1 score increase	*P* for trend *	*P* for interaction
	Quartile 1	Quartile 2	Quartile 3	Quartile 4			
	29.5–69.6	69.6–76.0	76.0–81.4	81.4–98.3			
**Total, n**	5461	5461	5461	5461			
Case number, n (%)	1758 (32.19)	928 (16.99)	576 (10.55)	248 (4.54)			
Incidence rate per 1,000	132.54	65.58	40.38	17.76			
Model 1	1.00 (Reference)	0.59 (0.54–0.63)	0.40 (0.36–0.44)	0.19 (0.16–0.21)	0.95 (0.94–0.95)	< 0.001	
Model 2	1.00 (Reference)	0.58 (0.53–0.63)	0.39 (0.35–0.43)	0.18 (0.15–0.20)	0.94 (0.94–0.95)	< 0.001	
Model 3	**1.00 (Reference)**	**0.69 (0.63–0.75)**	**0.50 (0.45–0.56)**	**0.24 (0.21–0.28)**	**0.96 (0.95–0.97)**	** < 0.001**	
**Sex**							0.127
** Female**	1306	3054	3924	4556			
Case number, n (%)	343 (26.26)	424 (13.88)	348 (8.87)	182 (3.99)			
Incidence rate per 1,000	97.81	50.70	33.01	15.45			
Model 3	**1.00 (Reference)**	**0.72 (0.62–0.84)**	**0.53 (0.45–0.62)**	**0.27 (0.22–0.32)**	**0.94 (0.93–0.95)**	** < 0.001**	
Male	4155	2407	1537	905			
Case number, n (%)	1415 (34.06)	504 (20.94)	228 (14.83)	66 (7.29)			
Incidence rate per 1,000	145.03	87.08	61.25	30.24			
Model 3	**1.00 (Reference)**	**0.68 (0.61–0.75)**	**0.51 (0.44–0.59)**	**0.26 (0.20–0.33)**	**0.97 (0.96–0.97)**	** < 0.001**	
**Age, year**							0.157
< 40	2767	3389	3602	3585			
Case number, n (%)	852 (30.79)	497 (14.67)	310 (8.61)	135 (3.77)			
Incidence rate per 1,000	129.94	56.44	33.23	15.30			
Model 3	**1.00 (Reference)**	**0.71 (0.63–0.79)**	**0.52 (0.45–0.60)**	**0.29 (0.23–0.35)**	**0.96 (0.95–0.97)**	** < 0.001**	
≥ 40	2694	2072	1859	1876			
Case number, n (%)	906 (33.63)	431 (20.80)	266 (14.31)	113 (6.02)			
Incidence rate per 1,000	135.09	80.62	53.89	21.98			
Model 3	**1.00 (Reference)**	**0.70 (0.63–0.79)**	**0.53 (0.45–0.61)**	**0.22 (0.18–0.27)**	**0.96 (0.96–0.97)**	** < 0.001**	

Model 1 was adjusted for age (years), sex. Model 2 was adjusted for model 1 plus education level (high school or lower, or university/college or above) and drinking status (none, mild, moderate). Model 3 was adjusted for model 2 plus waist circumference, eGFR, ALT at exam1, and antidiabetic, lipid-lowering, or antihypertensive medications usage before Exam4

^*^Test for trend based on variable containing median value for each quartile

Table 3 presents the adjusted HRs of incident NAFLD associated with quartiles of baseline (Exam 1) LE8 exposure. In fully adjusted model 3, compared with participants exposed to the lowest quartile, those exposed to the highest quartile had a 42% lower risk for incident NAFLD. For every 1 score increase in baseline LE8, the risk of incident NAFLD decreased by 1% (HR 0.99, 95% CI 0.98–0.99). A similar effect was observed across sex and age subgroups.

**Table 3:** Risks of NAFLD Development according to the baseline (exam 1) LE8 (*n* = 21,844)

	Groups of baseline-LE8 exposure				1 score increase	*P* for trend*	*P* for interaction
	Quartile 1	Quartile 2	Quartile 3	Quartile 4			
	29.5–69.6	69.6–76.0	76.0–81.4	81.4–98.3			
**Total, n**	6267	3630	4680	7267			
Case number, n (%)	1540 (24.57)	714 (19.67)	641 (13.70)	615(8.46)			
Incidence rate per 1,000	98.51	77.69	53.00	32.84			
Model 1	1.00 (Reference)	0.82 (0.75–0.90)	0.63 (0.57–0.69)	0.44 (0.40–0.49)	0.98 (0.97–0.98)	< 0.001	
Model 2	1.00 (Reference)	0.81 (0.74–0.89)	0.62 (0.57–0.68)	0.43 (0.39–0.48)	0.97 (0.97–0.98)	< 0.001	
Model 3	**1.00 (Reference)**	**0.87 (0.80–0.96)**	**0.76 (0.69–0.83)**	**0.58 (0.53–0.65)**	**0.99 (0.98–0.99)**	** < 0.001**	
**Sex**							
** Female**	2551	1866	2977	5446			0.098
Case number, n (%)	435 (17.05)	251 (13.45)	268 (9.00)	343 (6.30)			
Incidence rate per 1,000	62.82	51.15	33.65	23.82			
Model 3	**1.00 (Reference)**	**0.88 (0.76–1.03)**	**0.70 (0.60–0.82)**	**0.59 (0.51–0.69)**	**0.98 (0.98–0.99)**	** < 0.001**	
** Male**	3716	1764	1703	1821			
Case number, n (%)	1105 (29.74)	463 (26.25)	373 (21.90)	272 (14.94)			
Incidence rate per 1,000	126.90	108.07	90.34	62.83			
Model 3	**1.00 (Reference)**	**0.85 (0.77– 0.96)**	**0.80 (0.71–0.90)**	**0.61 (0.53–0.70)**	**0.99 (0.98–0.99)**	** < 0.001**	
**Age, year**							0.076
** < 40**	3212	1983	2962	5186			
Case number, n (%)	698 (21.73)	341 (17.20)	358 (12.09)	397 (7.66)			
Incidence rate per 1,000	89.62	71.108	47.19	29.74			
Model 3	**1.00 (Reference)**	**0.86 (0.75– 0.98)**	**0.76 (0.67–0.87)**	**0.62 (0.54–0.71)**	**0.98 (0.98–0.99)**	** < 0.001**	
** ≥ 40**	3055	1647	1718	2081			
Case number, n (%)	842 (27.56)	373 (22.65)	283 (16.47)	218 (10.48)			
Incidence rate per 1,000	107.34	84.86	62.79	40.50			
Model 3	**1.00 (Reference)**	**0.88 (0.77– 0.99)**	**0.75 (0.66–0.88)**	**0.58 (0.49–0.68)**	**0.99 (0.98–0.99)**	** < 0.001**	

Model 1 was adjusted for age (years), sex. Model 2 was adjusted for model 1 plus education level (high school or lower, or university/college or above) and drinking status (none, mild, moderate). Model 3 was adjusted for model 2 plus waist circumference, eGFR, ALT at exam1, and antidiabetic, lipid-lowering, or antihypertensive medications usage before Exam4

^*^ Test for trend based on variable containing median value for each quartile

### Association Between LE8 Exposure and NAFLD Regression

Table 4 presents the regression of NAFLD according to quartiles of cum-LE8 exposure in subjects with existing NAFLD at baseline. During a median follow-up of 2.4 (IQR, 1.2–3.2) years, 469 (27.6 per 1,000 person-years) subjects experienced NAFLD regression from Exam 4 to the last exam. The HRs of NAFLD regression increased steadily as cumLE8 exposure increased. The adjusted HRs (95% CI) for the regression of NAFLD comparing participants in quartiles 2, 3 and 4 to those in quartile 1 were 1.21 (0.88–1.64), 1.51 (1.12–2.04) and 2.03 (1.51–2.74), respectively. For every 1 score increase in cum-LE8, the risk of NAFLD regression increased by 3% (HR 1.03, 95% CI 1.02–1.04) in a dose‒response relationship (*P* for trend < 0.001). Similar results were found across age and sex subgroups.

**Table 4:** Risks of NAFLD regression according to the cumulative exposure of LE8 (*n* = 6,566)

	Groups of cum-LE8 exposure				1 score increase	*P* for trend*	*P* for interaction
	Quartile 1	Quartile 2	Quartile 3	Quartile 4			
	26.2–53.4	53.4–60.4	60.4–67.2	67.2–92.7			
**Total, n**	1641	1642	1642	1641			
Case number, n (%)	72 (4.39)	96 (5.85)	130 (7.92)	171 (10.42)			
Incidence rate per 1,000	17.3	22.5	29.2	41.1			
Model 1	1.00 (Reference)	1.27 (0.93–1.72)	1.61 (1.20–2.15)	2.23 (1.68–2.97)	1.04 (1.03–1.05)	< 0.001	
Model 2	1.00 (Reference)	1.26 (0.92–1.71)	1.60 (1.19–2.14)	2.24 (1.69–2.98)	1.03 (1.02–1.04)	< 0.001	
Model 3	**1.00 (Reference)**	**1.21 (0.88–1.64)**	**1.51 (1.12–2.04)**	**2.03 (1.51–2.74)**	**1.03 (1.02–1.04)**	** < 0.001**	
**Sex**							
** Female**	48	148	222	446			0.127
Case number, n (%)	2 (4.17)	8 (5.41)	17 (7.66)	63 (14.13)			
Incidence rate per 1,000	14.89	20.30	26.86	56.74			
Model 3	**1.00 (Reference)**	**1.38 (0.29–6.49)**	**1.98 (0.45–8.63)**	**4.32 (1.04–17.99)**	**1.04 (1.01–1.07)**	**0.001**	
** Male**	1593	1494	1420	1195			
Case number, n (%)	70 (4.39)	88 (5.89)	113 (7.96)	108 (9.04)			
Incidence rate per 1,000	17.42	22.79	29.59	35.46			
Model 3	**1.00 (Reference)**	**1.20 (0.88–1.66)**	**1.52 (1.12–2.06)**	**1.76 (1.28–2.42)**	**1.03 (1.02–1.04)**	** < 0.001**	
**Age, year**							0.602
** < 40**	775	697	660	599			
Case number, n (%)	37 (4.77)	52 (7.46)	55 (8.33)	66 (11.02)			
Incidence rate per 1,000	19.81	29.22	31.65	47.39			
Model 3	**1.00 (Reference)**	**1.38 (0.90–2.11)**	**1.43 (0.93–2.20)**	**2.00 (1.29–3.11)**	**1.03 (1.02–1.05)**	** < 0.001**	
** ≥ 40**	866	945	982	1042			
Case number, n (%)	35 (4.04)	44 (4.66)	75 (7.64)	105 (10.08)			
Incidence rate per 1,000	15.32	17.77	27.64	38.00			
Model 3	**1.00 (Reference)**	**1.02 (0.65–1.60)**	**1.42 (0.94–2.15)**	**1.85 (1.22–2.80)**	**1.03 (1.01–1.04)**	** < 0.001**	

Model 1 was adjusted for age (years), sex. Model 2 was adjusted for model 1 plus education level (high school or lower, or university/college or above) and drinking status (none, mild, moderate). Model 3 was adjusted for model 2 plus waist circumference, eGFR, ALT at exam1, and antidiabetic, lipid-lowering, or antihypertensive medications usage before Exam4

^*^ Test for trend based on variable containing median value for each quartile

Table 5 presents the adjusted HRs of NAFLD regression associated with quartiles of baseline (Exam 1) LE8 exposure. As the baseline LE8 score increased, the risk of NAFLD regression increased. The highest quartile exhibited a 61% higher risk for NAFLD regression compared with participants exposed to the lowest quartile. For every 1 score increase in baseline LE8, the risk of NAFLD regression increased by 1% (HR 1.01, 95% CI 1.01–1.03). Similar findings were demonstrated in subgroup analyses, except females with higher risks of nonstatistical significance.

**Table 5:** Risks of NAFLD regression according to the baseline (exam 1) exposure of LE8 (*n* = 6,566)

	Groups of baseline-LE8 exposure				1 score increase	*P* for trend*	*P* for interaction
	Quartile 1	Quartile 2	Quartile 3	Quartile 4			
	26.2–53.4	53.4–60.4	60.4–67.2	67.2–92.7			
**Total, n**	2044	1540	1420	1562			
Case number, n (%)	118 (5.77)	102 (6.62)	107 (7.54)	142 (9.09)			
Incidence rate per 1,000	21.51	24.80	29.02	38.07			
Model 1	1.00 (Reference)	1.17 (0.90–1.52)	1.36 (1.04–1.76)	1.78 (1.39–2.28)	1.02 (1.01–1.03)	< 0.001	
Model 2	1.00 (Reference)	1.16 (0.89–1.51)	1.35 (1.03–1.75)	1.77 (1.38–2.27)	1.02 (1.01–1.03)	< 0.001	
Model 3	**1.00 (Reference)**	**1.14 (0.87–1.49)**	**1.28 (0.98–1.68)**	**1.61 (1.24–2.10)**	**1.01 (1.01–1.03)**	** < 0.001**	
**Sex**							
** Female**	139	158	193	374			0.342
Case number, n (%)	10 (7.19)	13(8.23)	21(10.88)	46 (12.30)			
Incidence rate per 1,000	22.47	27.73	43.90	52.31			
Model 3	**1.00 (Reference)**	**1.21 (0.52–2.80)**	**1.81 (0.83–3.96)**	**2.05 (0.98–4.26)**	**1.02 (0.99–1.04)**	**0.026**	
** Male**	1905	1382	1227	1188			
Case number, n (%)	108 (5.67)	89 (6.44)	86 (7.01)	96 (8.08)			
Incidence rate per 1,000	21.43	24.42	26.81	33.68			
Model 3	**1.00 (Reference)**	**1.14 (0.86–1.52)**	**1.19 (0.89–1.60)**	**1.53 (1.14–2.05)**	**1.02 (1.01–1.03)**	**0.001**	
**Age, year**							0.594
** < 40**	784	657	609	681			
Case number, n (%)	43 (5.48)	43 (6.54)	50 (8.21)	74 (10.87)			
Incidence rate per 1,000	21.89	25.21	32.27	47.47			
Model 3	**1.00 (Reference)**	**1.11 (0.73–1.71)**	**1.30 (0.85–1.98)**	**1.91 (1.27–2.86)**	**1.02 (1.01–1.03)**	**0.001**	
** ≥ 40**	1260	883	811	881			
Case number, n (%)	75 (5.95)	59 (6.68)	57 (7.03)	68 (7.72)			
Incidence rate per 1,000	21.30	24.50	26.67	31.32			
Model 3	**1.00 (Reference)**	**1.15 (0.82–1.63)**	**1.27 (0.89–1.81)**	**1.53 (1.08–2.17)**	**1.01 (0.99–1.03)**	**0.015**	

Model 1 was adjusted for age (years), sex. Model 2 was adjusted for model 1 plus education level (high school or lower, or university/college or above) and drinking status (none, mild, moderate). Model 3 was adjusted for model 2 plus waist circumference, eGFR, ALT at exam1, and antidiabetic, lipid-lowering, or antihypertensive medications usage before Exam4

^*^Test for trend based on variable containing median value for each quartile

### Sensitivity analysis

Our study results were consistent across all sensitivity analyses, including (1) when the cum-health behaviors and cum-health factors were performed separately, the associations were unaffected (Supplementary Fig. 1 and Fig. 2). (2) After applying the 3 levels of the CVH score (low: LE8 < 50, moderate: 50 ≤ LE8 < 80, high: LE8 ≥ 80), similar results were yielded for the associations of cum- or baseline-LE8 and incident NAFLD with the decreased risks attenuated across increasing CVH groups (Supplementary Table 5 and Table 6). On the other hand, for NAFLD regressed to non-NAFLD, similar associations were found for cum-LE8 but not for baseline-LE8, which could be limited by the relatively small sample size of cases in the high group (Supplementary Table 7 and Table 8). (3) Similar impacts of baseline LE8 on the risk of incident NAFLD and regression of existing NAFLD were found in subjects who only needed to attend the first follow-up examination (Exam 2) (Supplementary Table 9 and Table 10).

## Discussion

In this large cohort study from Hunan, China, we confirmed that greater baseline and cumulative exposure to ideal cardiovascular health defined by the new LE8 metrics were associated with a markedly lower risk of NAFLD development and a higher beneficial effect of NAFLD regression among health check-up adults. Similar associations were observed across sex and age subgroups and were robust after adjustment for major covariates and through several sensitivity analyses. Furthermore, it is noteworthy that the cumulative exposure effect was greater than just the baseline level at a single time point. These findings suggest that promoting and preserving high CVH may yield benefits related to promoting hepatic health.

Multiple epidemiologic studies have assessed the association between LS7 and NAFLD among different racial populations, and we have summarized the similar literature in Supplementary Table 11. These findings revealed that achieving ideal CVH metrics could lead to favorable liver health. However, as the predecessor of LE8, LS7 feature definitions may not be able to reflect the full scope of health behaviors and practices and may be less sensitive to interindividual differences. After LE8 was proposed in 2022, three studies explored the relationship between LE8 and NAFLD. A cross-sectional study of 3,588 US adults found a negative association between LE8 scores and the burden of NAFLD [9]. Another study in the United States reported strong protective associations of LE8 with MAFLD as well as clinically significant fibrosis in individuals with MAFLD among 1,812 individuals [19]. The two studies were limited by small sample sizes, and both were cross-sectional designs that could not conclude causality. Recently, He et al. conducted a prospective study and found that a favorable lifestyle and a higher LE8 score were significantly associated with a lower risk of new-onset severe NAFLD in UK Biobank of 266,645 participants with a median follow-up of 11.9 years [20]. However, this study evaluated NAFLD risk based on only a single measure of CVH exposure at baseline. The CVH metrics were modifiable health behaviors and factors, and a single measurement approach could not distinguish between individuals who maintained poor CVH status over a long time and those who deteriorated to a low CVH score in a short period. As such, the relationship between CVH and the risk of NAFLD development was likely to be underestimated. In our study, the relationship was well characterized based on both baseline and cumulative measures and in bidirectional disease courses of NAFLD development and regression. To our knowledge, our findings provide the first evidence that increasing chronic exposure to ideal health behaviors and factors is not only strongly related to favorable prevention and treatment of NAFLD but that it is likely to more accurately reflect the true magnitude of risks compared with a single measurement.

The biological mechanisms underlying the correlation between CVH and NAFLD development and regression remain to be elucidated. CVH metrics have been shown to participate in the pathogenesis of NAFLD involving insulin resistance, abnormal lipoprotein metabolism, chronic low-grade inflammation, excessive oxidative stress, adipose tissue dysfunction and hepatic de novo lipogenesis, endothelial dysfunction, dysbiosis of the gut microbial ecology and epigenetics. As expected, there was a high burden of cardiovascular metabolic comorbidities associated with NAFLD. Obesity was present in 51% of individuals with NAFLD [21, 22]. Diabetes mellitus was identified in 23% of NAFLD cases [22]. The prevalence of metabolic syndrome and hyperlipidemia/dyslipidemia was 46.4% and 69% among NAFLD subjects, respectively [22, 23]. Nonalcoholic steatohepatitis (NASH) and atherosclerosis were suggested as two aspects of a shared disease [24]. Lifestyle interventions, including changes in dietary patterns, weight reduction, and physical exercise, are recommended as the cornerstone therapy by guidelines and expert consensus statements for NAFLD management. Several controlled clinical trials (RCTs) have demonstrated that lifestyle interventions (exercise alone or combined with dietary change) may have beneficial effects on reduced liver fat and metabolic profiles [25]. It is therefore not surprising that a composite score of all the LE8 metrics is associated with NAFLD. Taken together, CVH is uniquely positioned as the result of upstream genetic, social and environmental factors and the risks of major downstream health outcomes on the disease chain across the life course.

Thus, tracking CVH over time is crucial for NAFLD prevention and treatment. It is never too late to receive welfare from improvement in CVH [26]. The earlier that CVH is improved, the better the health outcomes are. Advances in electronic health (eHealth) technology (e.g., online websites, apps or WeChat) could be applied to facilitate CVH monitoring for assessing CVH status, recording lifestyle interventions and tracking its dynamic progress because of their high accessibility and affordability.

To our knowledge, this is the first study of the relationship between cumulative exposure to the new LE8 metrics and NAFLD development or regression in the Chinese population. Our study has several noteworthy advantages, including longitudinal design, large NAFLD sample in a well-characterized population, harmonized data set with multiple examinations, rich covariable adjustments encompassing sociodemographic factors and a series of sensitivity analyses that added robustness to our findings. Meanwhile, some limitations should be noted. First, our results might not be generalizable for a nationally representative population because of selection bias by excluding individuals who did not receive 4 consecutive annual health examinations and by only including our single-center data. Moreover, the study populations were mostly services employees and workers -derived but do not represent random samples, and study data may not necessarily apply to common populations of Chinese heritage. Second, one’s ability to choose healthy lifestyles across the life course is strongly influenced by psychological health factors and social and structural determinants; however, our study did not include psychological factors. Third, although ultrasonography is widely (applied in 90.56% of all NAFLD-related studies in China) and accurately (pooled sensitivity, 84.8%; specificity, 93.6%) performed to detect fatty liver due to invasiveness considerations, this could lead to potential false negative results [3]. Fourth, a self-administered questionnaire was used to calculate the health behavior of the LE8 score, which may have introduced recall bias. Fifth, due to the overlap of the components of LE8 and the definition of MAFLD, we selected NAFLD instead of MAFLD as the research objective. Last, there is a substantial loss to follow-up between exam 4 and 5, which may bring a systemic bias in the analysis of cumulative exposure to LE8 quality.

## Conclusion

This cohort study provides evidence that meeting high levels of CVH may be associated with a reduced future burden of NAFLD by minimizing the risk of incidence and improving remission. The study also highlights the importance of accounting for maintaining or adopting an ideal CVH while assessing risk rather than reliance on a single measure of CVH. The implementation of CVH improvement strategies should be organically incorporated into national health policies and health-care systems for NAFLD.

### Supplementary Information

Supplementary Material 1.

## Data Availability

No datasets were generated or analysed during the current study.
