# Supplementary material for: Cumulative exposure to AHA Life's Essential 8 is associated with nonalcoholic fatty liver disease: a large cohort study
Source: Nutr Metab (Lond). 2024 Jun 27;21:38. doi: 10.1186/s12986-024-00821-z (PMC11212352; doi:10.1186/s12986-024-00821-z)
Supplement: Supplementary file 1 — Supplementary Material 1. [file 12986_2024_821_MOESM1_ESM.docx]

Supplementary Material

**Supplementary Item 1:** Detailed information on the laboratory analysis.

**Supplementary Item 2:** Definition for covariates.

**Supplementary Table 1.** Definition and scoring approach Life’s Essential 8 score **Supplementary Table 2.** Definition and quantities for DASH score

**Supplementary Table 3.** Characteristics stratified by quartiles of cumulative Life’s Essential 8 scores in NAFLD development cohort (n = 21,844).

**Supplementary Table 4.** Characteristics stratified by quartiles of quartiles of cumulative Life’s Essential 8 scores in NAFLD regression cohort (n = 6,566).

**Supplementary Table 5.** Risks of NAFLD development in relation to different levels of cumulative exposure of Life’s Essential 8 (n = 21,844).

**Supplementary Table 6.** Risks of NAFLD development in relation to different levels of baseline (exam 1) exposure of Life’s Essential 8 (n = 21,844).

**Supplementary Table 7.** Risks of NAFLD regression in relation to different levels of cumulative exposure of Life’s Essential 8 (n = 6,566).

**Supplementary Table 8.** Risks of NAFLD regression in relation to different levels of baseline exposure of Life’s Essential 8 (n = 6,566).

**Supplementary Table 9.** Risks of NAFLD development (exam2) in relation to quartile increase of baseline (exam 1) exposure Life’s Essential 8 (n = 28,502).

**Supplementary Table 10.** Risks of NAFLD regression (exam2) in relation to quartile increase of baseline (exam 1) exposure Life’s Essential 8 (n = 7,452).

**Supplementary Table 11.** Previous studies of the relationship between ideal cardiovascular health metrics and risk of NAFLD transitions.

**Supplementary Figure 1.** Risks of NAFLD development in relation to quartile increase in cumulative exposure of health behaviors (diet, physical activity, nicotine exposure, and sleep duration) and ideal health factors (BMI, non– HDL– C, blood glucose, and blood pressure) (n = 21,844).

**Supplementary Figure 2.** Risks of NAFLD regression in relation to quartile increase in cumulative exposure of health behaviors (diet, physical activity, nicotine exposure, and sleep duration) and ideal health factors (BMI, non– HDL– C, blood glucose, and blood pressure) (n = 6,566).

**Supplementary Item 1: Detailed information on the laboratory analysis.**

The sample analysis was performed in accordance with the manufacturer’s specifications. Fasting blood glucose (FBG) was measured with the hexokinase/glucose– 6– phosphate– dehydrogenase method (intra– assay coefficient of variation, 2.5%). Total cholesterol (TC), high– density lipoprotein cholesterol (HDL– C), and triglycerides were measured with enzymatic methods. Low– density lipoprotein cholesterol (LDL– C) was measured directly[1]. Serum creatinine was measured enzymatically and alanine transaminase (ALT) was determined by the bromocresol green (BCG) method.

**Supplementary Item 2: Definition for covariates**

The educational attainment was categorized as “high school or lower” and “university/college or above”. The amount of alcohol consumed was assessed by the number of days consumed alcohol per week and the number of standard drinks per drinking session. In China, the most popular and commonly consumed alcoholic drinks are Chinese wine (1 cup = 12.5 g alcohol) and beer (1 cup = 7.8 g alcohol). The participants were divided into 4 categories: none; mild (>0 and <105 g per week); moderate (≥105 and <210 g per week); and heavy (≥210 g per week)[2]. Subjects with heavy alcoholic intake were excluded from the study.

Hypertension was defined as either a self– reported history of hypertension, SBP or DBP ≥ 140/90 mmHg, or specific antihypertensive treatment[3]. Diabetes was defined as self– reported history of diabetes, FBG ≥ 7.0 mmol/L, use of insulin or oral anti– diabetic drugs[4]. Dyslipidemia was defined as the presence of one or more of the following: total cholesterol (TC) ≥ 6.22 mmol/L; low– density lipoprotein cholesterol (LDL– C) ≥ 4.14 mmol/L; density lipoprotein cholesterol (HDL– C) <1.04 mmol/L; triglyceride (TG) ≥ 2.26 mmol/L, or treatment for dyslipidemia[5]. Individuals were considered to have a history of CVD if they self– reported a physician– diagnosed stroke or physician– diagnosed coronary heart disease (CHD). Individuals were considered to have a history of chronic kidney disease (CKD) if they self– reported a physician– diagnosed CKD or eGFR <60 mL/min/1.73 m^2^[6].

**Supplementary Table 1. Definition and scoring approach Life’s Essential 8 score[7].**

| **Domain** | **CVH Metric** | **Measurement** | **Quantification and Scoring of CVH Metric** |
| --- | --- | --- | --- |
| Health Behaviors | 1.Diet | Dietary Approaches to Stop (DASH) score percentile | Quantiles of DASH– style diet adherence  **Scoring (Population):**  Points Quantile  100 ≥95^th^ percentile  80 75^th^ – 94^th^ percentile  50 50^th^ – 74^th^ percentile  25 25^th^ – 49^th^ percentile  0 1^st^ – 24^th^ percentile |
|  | 2.Physical activity | Self– reported minutes of moderate or vigorous physical activity per week | **Metric:** Minutes of moderate (or greater) intensity activity per week  **Scoring:**  Points Minutes  100 ≥150  90 120 – 149  80 90 – 119  60 60 – 89  40 30 – 59  20 1 – 29  0 0 |
|  | 3.Nicotine exposure | Self– reported use of cigarettes or inhaled nicotine– delivery system | **Metric:** Combustible tobacco use and/or inhaled NDS use; or secondhand smoke exposure  **Scoring:**  Points Status  100 Never smoker  75 Former smoker, quit ≥5 years  50 Former smoker, quit 1 – <5 years  25 Former smoker, quit <1 year, or currently using inhaled NDS  0 Current smoker  Subtract 20 points (unless score is 0) for living with active indoor smoker in home |
|  | 4.Sleep health | Self– reported average hours of sleep per night | **Metric:** Average hours of sleep per night  **Scoring:**  Points Level  100 7 – <9  90 9 – <10  70 6 – <7  40 5 – <6 or ≥10  20 4 – <5  0 <4 |
| Health Factors | 5.Body mass index | Body weight (kg) divided by height squared (m^2^) | **Metric:** Body mass index (kg/m^2^)  **Scoring:**  Points Level  100 <25  70 25.0 – 29.9  30 30.0 – 34.9  15 35.0 – 39.9  0 ≥40.0 |
|  | 6.Blood lipids | Plasma total and HDL– cholesterol with calculation of non– HDL– cholesterol | **Metric:** Non– HDL– cholesterol (mmol/L)  **Scoring:**  Points Level  100 3.4  60 3.4 – 4.1  40 4.1 – 4.9  20 4.9 – 5.7  0 ≥5.7  If drug– treated level, subtract 20 points |
|  | 7.Blood glucose | Fasting blood glucose  The HbA1c were not available in our data, so the conversion formula of HBA1C to FBG (mg/L) was used: 28.7×HbA1c–46.7= FBG[8]  FBG unit conversion: 1mg = 0.056mmol/L, 1mmol/L= 18.02 mg/dL. | **Metric:** Fasting blood glucose (mmol/L)  **Scoring:**  Points Level  100 No history of diabetes and FBG <5.6  60 No diabetes and FBG 5.6 – 6.9 (Pre– diabetes)  40 Diabetes with FBG <8.6  30 Diabetes with FBG 8.6 – 10.1  20 Diabetes with FBG 10.2 – 11.6  10 Diabetes with FBG 11.7 – 13.2  0 Diabetes with FBG ≥13.3 |
|  | 8.Blood pressure | Appropriately measured systolic and diastolic blood pressure | **Metric:** Systolic and diastolic blood pressure (mm Hg)  **Scoring:**  Points Level  100 <120/<80 (Optimal)  75 120– 129/<80 (Elevated)  50 130– 139 or 80– 89 (Stage I HTN)  25 140– 159 or 90– 99  0 ≥160 or ≥100  Subtract 20 points if treated level |

**Supplementary Table 2. Definition and quantities for DASH score[9]**

|  | **DASH** |  |
| --- | --- | --- |
| **Component** | **Criteria for 5 points** | **Criteria for 1 point** |
| Vegetables (excluding potatoes and legumes) | highest quintile | Lowest quintile |
| Total fruit | highest quintile | Lowest quintile |
| Nut and legumes | Highest quintile | Lowest quintile |
|  |  |  |
| Whole grains | Highest quintile | Lowest quintile |
| Dairy | Highest quintile | Lowest quintile |
| Red and processed meat | Lowest quintile | Highest quintile |
| SSBs and fruit juice | Lowest quintile | Highest quintile |
| Sodium | Lowest quintile | Highest quintile |
| Total | 40 |  |

Abbreviations: DASH: Dietary Approaches to Stop Hypertension; SSB, Sugar-sweetened beverage.

**Supplementary Table 3. Characteristics stratified by quartiles of cumulative Life’s Essential 8 scores in NAFLD development cohort (n = 21,844).**

| **Characteristics** | **Groups of cum-LE8 exposure** | | | | ***P* value** |
| --- | --- | --- | --- | --- | --- |
|  | Quartile 1 | Quartile 2 | Quartile 3 | Quartile 4 |  |
|  | 29.5– 69.6 | 69.6– 76.0 | 76.0– 81.4 | 81.4– 98.3 |  |
| Total, n | 5461 | 5461 | 5461 | 5461 |  |
| Age, mean (SD), y | 42.5 (13.5) | 39.3 (13.1) | 38.1 (1.2) | 37.5 (10.8) | < 0.001 |
| Female, n (%) | 1306 (23.9) | 3054 (55.9) | 3924 (71.9) | 4556 (83.4) | < 0.001 |
| University degree, n (%) | 4289 (78.5) | 4692 (85.9) | 4883 (89.4) | 4980 (91.2) | < 0.001 |
| BMI, kg/m^2^ | 23.1 (2.6) | 21.9 (2.4) | 21.4 (2.2) | 21.1 (2.0) | < 0.001 |
| WC, cm | 80.0 (7.6) | 75.2 (7.5) | 73.3 (6.9) | 71.8 (6.3) | < 0.001 |
| Systolic blood pressure, mm Hg | 124.0 (14.8) | 117.3 (13.8) | 113.3 (12.5) | 109.5 (10.7) | < 0.001 |
| Diastolic blood pressure, mm Hg | 76.4 (10.3) | 71.8 (9.3) | 69.6 (8.7) | 66.9 (7.6) | < 0.001 |
| Fasting glucose, mmol/L | 5.4 (1.2) | 5.1 (0.6) | 5.0 (0.5) | 4.9 (0.4) | < 0.001 |
| Non– HDL cholesterol, mmol/L | 3.5 (0.9) | 3.0 (0.8) | 2.8 (0.8) | 2.6 (0.7) | < 0.001 |
| ALT, U/L | 20.0 (15.0– 27.0) | 17.0 (13.0– 23.0) | 15.0 (12.0– 21.0) | 15.0 (12.0– 19.0) | < 0.001 |
| eGFR, mL/min/1.73m2 | 109.8 (96.2– 126.3) | 120.5 (104.1– 140.7) | 126.6 (108.8– 147.2) | 128.6 (111.5– 148.6) | < 0.001 |
| Hypertension, n (%) | 1179 (21.6) | 491 (9.0) | 268 (4.9) | 67 (1.2) | < 0.001 |
| Diabetes mellitus, n (%) | 316 (5.8) | 92 (1.7) | 38 (0.7) | 17 (0.3) | < 0.001 |
| Dyslipidemia, n (%) | 1244 (22.8) | 498 (9.1) | 311 (5.7) | 154 (2.8) | < 0.001 |
| Follow– up period (years) | 2.1 (1.0– 3.2) | 2.3 (1.2– 3.4) | 2.6 (1.3– 3.3) | 2.3 (1.2– 3.3) | < 0.001 |
| Times of screening exams | 5.1 (1.3) | 5.2 (1.3) | 5.2 (1.3) | 5.1 (1.3) | < 0.001 |

Values are n (%), mean ± SD, or median (first quartile, third quartile).

Abbreviations: BMI, Body mass index; WC, waist circumference; HDL, high– density lipoprotein; ALT, alanine aminotransferase; eGFR, estimated glomerular filtration rate.

**Supplementary Table 4. Characteristics stratified by quartiles of quartiles of cumulative Life’s Essential 8 scores in NAFLD regression cohort (n = 6,566).**

| **Characteristics** | **Groups of cum-LE8 exposure** | | | | ***P* value** |
| --- | --- | --- | --- | --- | --- |
|  | Quartile 1 | Quartile 2 | Quartile 3 | Quartile 4 |  |
|  | 26.2– 53.4 | 53.4– 60.4 | 60.4– 67.2 | 67.2– 92.7 |  |
| Total, n | 1641 | 1642 | 1642 | 1641 |  |
| Age, mean (SD), y | 41.8 (10.0) | 43.4 (10.8) | 44.9 (12.3) | 45.7 (12.3) | < 0.001 |
| Female, n (%) | 518 (9.6) | 206 (25.3) | 99 (37.6) | 41 (45.1) | < 0.001 |
| University degree, n (%) | 4572 (84.7) | 725 (89.1) | 232 (88.2) | 86 (94.5) | < 0.001 |
| BMI, kg/m^2^ | 27.9 (2.9) | 26.9 (2.4) | 26.3 (2.2) | 25.5 (2.3) | < 0.001 |
| WC, cm | 93.7 (7.3) | 91.0 (6.7) | 89.2 (6.5) | 86.7 (6.7) | < 0.001 |
| Systolic blood pressure, mm Hg | 130.6 (12.8) | 127.9 (13.1) | 126.4 (12.8) | 122.5 (11.9) | < 0.001 |
| Diastolic blood pressure, mm Hg | 83.5 (10.1) | 80.7 (9.7) | 79.1 (9.4) | 76.1 (8.8) | < 0.001 |
| Fasting glucose, mmol/L | 5.8 (1.6) | 5.6 (1.10) | 5. 5 (0.8) | 5.3 (0.7) | < 0.001 |
| Non– HDL cholesterol, mmol/L | 4.3 (0.9) | 3.9 (0.8) | 3.7 (0.8) | 3.4 (0.8) | < 0.001 |
| ALT, U/L | 39.0 (27.0– 58.0) | 36.0 (26.0– 52.0) | 33.0 (24.0– 48.0) | 30.0 (22.0– 43.0) | < 0.001 |
| eGFR, mL/min/1.73m2 | 109.6 (96.5– 124.2) | 108.1 (95.1– 122.9) | 107.0 (94.5– 122.0) | 108.9 (94.9– 126.0) | 0.016 |
| Hypertension, n (%) | 1465 (27.1) | 120 (14.7) | 26 (9.9) | 1 (1.1) | < 0.001 |
| Diabetes mellitus, n (%) | 404 (7.5) | 21 (2.6) | 5 (1.9) | 4 (4.4) | < 0.001 |
| Dyslipidemia, n (%) | 2753 (51.0) | 260 (31.9) | 60 (22.8) | 18 (19.8) | < 0.001 |
| Follow– up period (years) | 2.16 (1.13–3.17) | 2.33 (1.17– 3.25) | 2.83 (1.67– 3.25) | 2.33 (1.17– 3.17) | < 0.001 |
| Times of screening exams | 5.0 (1.3) | 5.2 (1.4) | 5.4 (1.5) | 5.3 (1.5) | < 0.001 |

Values are n (%), mean ± SD, or median (first quartile, third quartile).

Abbreviations: BMI, Body mass index; WC, waist circumference; HDL, high– density lipoprotein; ALT, alanine aminotransferase; eGFR, estimated glomerular filtration rate.

**Supplementary Table 5.** **Risks of NAFLD development in relation to different levels of cumulative exposure of Life’s Essential 8 (n = 21,844).**

|  | **Groups of cum-LE 8 exposure** | | | **P for trend*** | **P for interaction** |
| --- | --- | --- | --- | --- | --- |
|  | Low CVH | Moderate CVH | High CVH |  |  |
|  | 0– 49 | 50– 79 | 80– 100 |  |  |
| Total, n | 209 | 14870 | 6765 |  |  |
| Case number, n (%) | 111 (53.1) | 3041 (20.5) | 358 (5.3) |  |  |
| Incidence rate per 1,000 | 237.9 | 80.4 | 20.6 |  |  |
| Model 1 | 1.00 (Reference) | 0.46 (0.38– 0.56) | 0.16 (0.13– 0.19) | < 0.001 |  |
| Model 2 | 1.00 (Reference) | 0.45 (0.37– 0.55) | 0.15 (0.12– 0.18) | < 0.001 |  |
| Model 3 | **1.00 (Reference)** | **0.48 (0.40– 0.58)** | **0.16 (0.13– 0.20)** | **< 0.001** |  |
| **Sex** |  |  |  |  |  |
| **Female** | 5 | 7266 | 5569 |  | 0.968 |
| Case number, n (%) | 3 (60.0) | 1038 (14.3) | 256 (4.6) |  |  |
| Incidence rate per 1,000 | 158.6 | 52.7 | 17.7 |  |  |
| Model 3 | **1.00 (Reference)** | **0.58 (0.19– 1.82)** | **0.21 (0.07– 0.66)** | **< 0.001** |  |
| **Male** | 204 | 7604 | 1196 |  |  |
| Case number, n (%) | 108 (52.9) | 2003 (26.3) | 102 (8.5) |  |  |
| Incidence rate per 1,000 | 241.3 | 110.5 | 35.5 |  |  |
| Model 3 | **1.00 (Reference)** | **0.45 (0.37– 0.55)** | **0.14 (0.11– 0.19)** | **< 0.001** |  |
| **Age, year** |  |  |  |  | 0.081 |
| **< 40** | 75 | 8804 | 4464 |  |  |
| Case number, n (%) | 43 (57.3) | 1554 (17.7) | 197 (4.4) |  |  |
| Incidence rate per 1,000 | 287.6 | 69.8 | 17.7 |  |  |
| Model 3 | **1.00 (Reference)** | **0.36 (0.26– 0.49)** | **0.14 (0.10– 0.20)** | **< 0.001** |  |
| **≥ 40** | 134 | 6066 | 2301 |  |  |
| Case number, n (%) | 68 (50.7) | 1487 (24.5) | 161 (7.0) |  |  |
| Incidence rate per 1,000 | 214.5 | 95.6 | 25.7 |  |  |
| Model 3 | **1.00 (Reference)** | **0.54 (0.42– 0.70)** | **0.16 (0.12– 0.21)** | **< 0.001** |  |

Model 1 was adjusted for age (years), sex. Model 2 was adjusted for model 1 plus education level (high school or lower, or university/college or above) and drinking status (none, mild, moderate). Model 3 was adjusted for model 2 plus waist circumference, eGFR, ALT at exam1, and antidiabetic, lipid-lowering, or antihypertensive medications usage before Exam4.

^*^ Test for trend based on variable containing median value for each quartile.

**Supplementary Table 6. Risks of NAFLD development in relation to different levels of baseline (exam 1) exposure of Life’s Essential 8 (n = 21,844).**

|  | **Groups of baseline-LE 8 exposure** | | | **P for trend*** | **P for interaction** |
| --- | --- | --- | --- | --- | --- |
|  | Low CVH | Moderate CVH | High CVH |  |  |
|  | 0– 49 | 50–79 | 80–100 |  |  |
| Total, n | 518 | 12525 | 8801 |  |  |
| Case number, n (%) | 176 (34.0) | 2550 (20.4) | 784 (8.9) |  |  |
| Incidence rate per 1,000 | 137.9 | 80.0 | 34.9 |  |  |
| Model 1 | 1.00 (Reference) | 0.74 (0.63– 0.86) | 0.40 (0.34– 0.48) | < 0.001 |  |
| Model 2 | 1.00 (Reference) | 0.73 (0.63– 0.85) | 0.39 (0.33– 0.47) | < 0.001 |  |
| Model 3 | **1.00 (Reference)** | **0.78 (0.67– 0.92)** | **0.44 (0.37– 0.52)** | **< 0.001** |  |
| **Sex** |  |  |  |  |  |
| **Female** | 93 | 6317 | 6430 |  | 0.096 |
| Case number, n (%) | 26 (28.0) | 851 (13.5) | 420 (6.5) |  |  |
| Incidence rate per 1,000 | 99.2 | 49.7 | 25.0 |  |  |
| Model 3 | **1.00 (Reference)** | **0.84 (0.56– 1.26)** | **0.50 (0.33– 0.75)** | **< 0.001** |  |
| **Male** | 425 | 6208 | 2371 |  |  |
| Case number, n (%) | 150 (35,3) | 1699 (27.4) | 364 (15.4) |  |  |
| Incidence rate per 1,000 | 147.9 | 115.0 | 64.3 |  |  |
| Model 3 | **1.00 (Reference)** | **0.76 (0.64– 0.90)** | **0.43 (0.35– 0.52)** | **< 0.001** |  |
| **Age, year** |  |  |  |  | 0.566 |
| **< 40** | 112 | 7067 | 6164 |  |  |
| Case number, n (%) | 39 (34.8) | 1255 (17.8) | 500 (8.1) |  |  |
| Incidence rate per 1,000 | 157.8 | 71.3 | 31.9 |  |  |
| Model 3 | **1.00 (Reference)** | **0.74 (0.53– 1.03)** | **0.45 (0.32– 0.63)** | **< 0.001** |  |
| **≥ 40** | 406 | 5458 | 2637 |  |  |
| Case number, n (%) | 137 (33.7) | 1295 (23.7) | 284 (10.8) |  |  |
| Incidence rate per 1,000 | 133.1 | 90.6 | 41.8 |  |  |
| Model 3 | **1.00 (Reference)** | **0.78 (0.64– 0.93)** | **0.41 (0.33– 0.51)** | **< 0.001** |  |

Model 1 was adjusted for age (years), sex. Model 2 was adjusted for model 1 plus education level (high school or lower, or university/college or above) and drinking status (none, mild, moderate). Model 3 was adjusted for model 2 plus waist circumference, eGFR, ALT at exam1, and antidiabetic, lipid-lowering, or antihypertensive medications usage before Exam4.

^*^ Test for trend based on variable containing median value for each quartile.

**Supplementary Table 7.** **Risks of NAFLD regression in relation to different levels of cumulative exposure of Life’s Essential 8 (n = 6,566).**

|  | **Groups of cum-LE8 exposure** | | | **P for trend*** | **P for interaction** |
| --- | --- | --- | --- | --- | --- |
|  | Low CVH | Moderate CVH | High CVH |  |  |
|  | 0– 49 | 50–79 | 80– 100 |  |  |
| Total, n | 1049 | 5087 | 430 |  |  |
| Case number, n (%) | 49 (4.7) | 379 (7.5) | 41 (9.5) |  |  |
| Incidence rate per 1,000 | 18.39 | 28.43 | 40.11 |  |  |
| Model 1 | 1.00 (Reference) | 1.46 (1.08– 1.97) | 1.94 (1.26– 2.98) | < 0.001 |  |
| Model 2 | 1.00 (Reference) | 1.45 (1.07– 1.96) | 1.92 (1.25– 2.95) | 0.001 |  |
| Model 3 | **1.00 (Reference)** | **1.32 (0.97– 1.79)** | **1.61 (1.03– 2.52)** | **0.031** |  |
| **Sex** |  |  |  |  |  |
| **Female** | 21 | 682 | 161 |  | 0.796 |
| Case number, n (%) | 2 (9.5) | 67 (9.8) | 21 (13.0) |  |  |
| Incidence rate per 1,000 | 35.55 | 36.69 | 53.91 |  |  |
| Model 3 | **1.00 (Reference)** | **0.86 (0.21– 3.57)** | **1.16 (0.27– 5.06)** | **0.481** |  |
| **Male** | 1028 | 4405 | 269 |  |  |
| Case number, n (%) | 47 (4.6) | 312 (7.1) | 20 (7.4) |  |  |
| Incidence rate per 1,000 | 18.02 | 27.12 | 31.62 |  |  |
| Model 3 | **1.00 (Reference)** | **1.45 (1.06– 1.98)** | **1.80 (1.06– 3.07)** | **0.049** |  |
| **Age, year** |  |  |  |  | 0.755 |
| **< 40** | 490 | 2104 | 137 |  |  |
| Case number, n (%) | 26 (5.3) | 168 (8.0) | 16 (11.7) |  |  |
| Incidence rate per 1,000 | 22.26 | 31.71 | 51.17 |  |  |
| Model 3 | **1.00 (Reference)** | **1.24 (0.81– 1.91)** | **1.65 (0.84– 3.23)** | **0.148** |  |
| **≥ 40** | 559 | 2983 | 293 |  |  |
| Case number, n (%) | 23 (4.1) | 211 (7.1) | 25 (8.5) |  |  |
| Incidence rate per 1,000 | 15.37 | 26.27 | 35.24 |  |  |
| Model 3 | **1.00 (Reference)** | **1.31 (0.84– 2.04)** | **1.51 (0.82– 2.76)** | **0.177** |  |

Model 1 was adjusted for age (years), sex. Model 2 was adjusted for model 1 plus education level (high school or lower, or university/college or above) and drinking status (none, mild, moderate). Model 3 was adjusted for model 2 plus waist circumference, eGFR, ALT at exam1, and antidiabetic, lipid-lowering, or antihypertensive medications usage before Exam4.

^*^ Test for trend based on variable containing median value for each quartile.

**Supplementary Table 8. Risks of NAFLD regression in relation to different levels of baseline exposure of Life’s Essential 8 (n = 6,566).**

|  | **Groups of baseline-LE8 exposure** | | | **P for trend*** | **P for interaction** |
| --- | --- | --- | --- | --- | --- |
|  | Low CVH | Moderate CVH | High CVH |  |  |
|  | 0– 49 | 50– 79 | 80– 100 |  |  |
| **Total, n** | 1391 | 4760 | 415 |  |  |
| Case number, n (%) | 70 (5.0) | 378(7.9) | 21 (5.1) |  |  |
| Incidence rate per 1,000 | 18.70 | 30.43 | 24.67 |  |  |
| Model 1 | 1.00 (Reference) | 1.62 (1.25– 2.09) | 1.37 (0.84– 2.25) | 0.002 |  |
| Model 2 | 1.00 (Reference) | 1.61 (1.24– 2.08) | 1.35 (0.83– 2.22) | 0.003 |  |
| Model 3 | **1.00 (Reference)** | **1.50 (1.16– 1.96)** | **1.16 (0.70– 1.92)** | **0.063** |  |
| **Sex** |  |  |  |  |  |
| **Female** | 89 | 650 | 125 |  | 0.055 |
| Case number, n (%) | 3 (3.4) | 76 (11.7) | 11 (8.8) |  |  |
| Incidence rate per 1,000 | 10.59 | 43.63 | 44.65 |  |  |
| Model 3 | **1.00 (Reference)** | **3.87 (1.19– 12.60)** | **3.52 (0.93– 13.27)** | **0.066** |  |
| **Male** | 1302 | 4110 | 290 |  |  |
| Case number, n (%) | 67 (5.1) | 302 (7.3) | 10 (3.4) |  |  |
| Incidence rate per 1,000 | 19.37 | 28.28 | 16.54 |  |  |
| Model 3 | **1.00 (Reference)** | **1.39 (1.06– 1.83)** | **0.84 (0.43– 1.66)** | **0.247** |  |
| **Age, year** |  |  |  |  | 0.847 |
| **< 40** | 535 | 2022 | 174 |  |  |
| Case number, n (%) | 26 (4.9) | 175 (8.7) | 9 (5.2) |  |  |
| Incidence rate per 1,000 | 19.41 | 34.35 | 26.17 |  |  |
| Model 3 | **1.00 (Reference)** | **1.55 (1.01– 2.37)** | **1.00 (0.45– 2.22)** | **0.399** |  |
| **≥ 40** | 856 | 2738 | 241 |  |  |
| Case number, n (%) | 44 (5.1) | 203 (7.4） | 12 (5.0) |  |  |
| Incidence rate per 1,000 | 18.31 | 27.70 | 23.66 |  |  |
| Model 3 | **1.00 (Reference)** | **1.38 (0.98– 1.94)** | **1.24 (0.64– 2.41)** | **0.161** |  |

Model 1 was adjusted for age (years), sex. Model 2 was adjusted for model 1 plus education level (high school or lower, or university/college or above) and drinking status (none, mild, moderate). Model 3 was adjusted for model 2 plus waist circumference, eGFR, ALT at exam1, and antidiabetic, lipid-lowering, or antihypertensive medications usage before Exam4.

^*^ Test for trend based on variable containing median value for each quartile.

**Supplementary Table 9. Risks of NAFLD development (exam2) in relation to quartile increase of baseline (exam 1) exposure Life’s Essential 8 (n = 28,502).**

|  | **Quartiles of baseline-LE8 exposure** | | | | **1 score increase** | ***P* for trend*** | ***P* for interaction** |
| --- | --- | --- | --- | --- | --- | --- | --- |
|  | Quartile 1 | Quartile 2 | Quartile 3 | Quartile 4 |  |  |  |
|  | 29.5– 69.6 | 69.6– 76.0 | 76.0– 81.4 | 81.4– 98.3 |  |  |  |
| **Total, n** | 9373 | 4826 | 6018 | 8285 |  |  |  |
| Case number, n (%) | 1646 (17.56) | 555 (11.50) | 560 (9.31) | 344 (4.16) |  |  |  |
| Incidence rate per 1,000 | 123.70 | 85.80 | 69.11 | 30.69 |  |  |  |
| Model 1 | 1.00 (Reference) | 0.81 (0.74– 0.89) | 0.76 (0.69– 0.84) | 0.41 (0.36– 0.46) | 0.98 (0.97– 0.98) | < 0.001 |  |
| Model 2 | 1.00 (Reference) | 0.80 (0.72– 0.89) | 0.75 (0.68– 0.83) | 0.40 (0.35– 0.45) | 0.97 (0.97– 0.98) | < 0.001 |  |
| Model 3 | **1.00 (Reference)** | **0.81 (0.73– 0.89)** | **0.77 (0.70– 0.85)** | **0.42 (0.37– 0.47)** | **0.98 (0.97– 0.98)** | **< 0.001** |  |
| **Sex** |  |  |  |  |  |  |  |
| **Female** | 3322 | 2317 | 3607 | 6069 |  |  | 0.328 |
| Case number, n (%) | 348 (10.48) | 165 (7.12) | 208 (5.77) | 157 (2.59) |  |  |  |
| Incidence rate per 1,000 | 71.05 | 52.36 | 42.19 | 18.79 |  |  |  |
| Model 3 | **1.00 (Reference)** | **0.81 (0.68– 0.98)** | **0.79 (0.66– 0.94)** | **0.38 (0.31– 0.46)** | **0.97 (0.96– 0.98)** | **< 0.001** |  |
| **Male** | 6051 | 2509 | 2411 | 2216 |  |  |  |
| Case number, n (%) | 1298 (21.45) | 390 (15.54) | 352 (14.60) | 187 (8.44) |  |  |  |
| Incidence rate per 1,000 | 154.37 | 117.59 | 110.96 | 65.51 |  |  |  |
| Model 3 | **1.00 (Reference)** | **0.80 (0.71– 0.90)** | **0.78 (0.69– 0.88)** | **0.49 (0.42– 0.57)** | **0.98 (0.98– 0.99)** | **< 0.001** |  |
| **Age, year** |  |  |  |  |  |  | 0.265 |
| **< 40** | 4464 | 2528 | 3704 | 5844 |  |  |  |
| Case number, n (%) | 621(13.91) | 246 (9.73) | 283 (7.64) | 194 (3.32) |  |  |  |
| Incidence rate per 1,000 | 93.52 | 69.64 | 55.18 | 24.16 |  |  |  |
| Model 3 | **1.00 (Reference)** | **0.92 (0.80– 1.07)** | **0.88 (0.76– 1.02)** | **0.49 (0.42– 0.58)** | **0.98 (0.97– 0.99)** | **< 0.001** |  |
| **≥ 40** | 4909 | 2298 | 2314 | 2441 |  |  |  |
| Case number, n (%) | 1025 （20.88） | 309(13.45) | 277 (11.97) | 150 (6.15) |  |  |  |
| Incidence rate per 1,000 | 153.77 | 105.25 | 93.14 | 47.17 |  |  |  |
| Model 3 | **1.00 (Reference)** | **0.74 (0.65– 0.85)** | **0.71 (0.62– 0.81)** | **0.39 (0.33– 0.47)** | **0.98 (0.97– 0.98)** | **< 0.001** |  |

Model 1 was adjusted for age (years), sex. Model 2 was adjusted for model 1 plus education level (college and above or not) and drinking status (none, mild, moderate). Model 3 was adjusted for model 2 plus waist circumference, eGFR, ALT and antidiabetic, lipid–lowering, or antihypertensive medications usage at exam1.

^*^ Test for trend based on variable containing median value for each quarter.

**Supplementary Table 10. Risks of NAFLD regression (exam2) in relation to quartile increase of baseline (exam 1) exposure Life’s Essential 8 (n = 7,452).**

|  | | **Quartiles of baseline-LE8 exposure** | | | | **1 score increase** | ***P* for trend*** | ***P* for interaction** |
| --- | --- | --- | --- | --- | --- | --- | --- | --- |
|  | Quartile 1 | | Quartile 2 | Quartile 3 | Quartile 4 |  |  |  |
|  | 26.2– 53.4 | | 53.4– 60.4 | 60.4– 67.2 | 67.2– 92.7 |  |  |  |
| **Total, n** | 2163 | | 1764 | 1716 | 1809 |  |  |  |
| Case number, n (%) | 48 (2.22) | | 85 (4.82) | 96 (5.59) | 127 (7.02) |  |  |  |
| Incidence rate per 1,000 | 16.04 | | 36.25 | 43.73 | 54.81 |  |  |  |
| Model 1 | 1.00 (Reference) | | 1.20 (0.92– 1.57) | 1.47 (1.31– 1.91) | 1.91 (1.49– 2.45) | 1.04 (1.03– 1.05) | < 0.001 |  |
| Model 2 | 1.00 (Reference) | | 2.18 (1.53– 3.11) | 2.68 (1.89– 3.80) | 3.31 (2.36– 4.65) | 1.04 (1.02– 1.05) | < 0.001 |  |
| Model 3 | **1.00 (Reference)** | | **2.06 (1.45– 2.95)** | **2.45 (1.72– 3.47)** | **2.88 (2.03– 4.08)** | **1.03 (1.02– 1.04)** | **< 0.001** |  |
| **Sex** |  | |  |  |  |  |  |  |
| **Female** | 152 | | 189 | 253 | 453 |  |  | 0.819 |
| Case number, n (%) | 5 (3.29） | | 12 (6.35) | 20 (7.91) | 46 (10.15) |  |  |  |
| Incidence rate per 1,000 | 25.49 | | 45.60 | 62.18 | 79.14 |  |  |  |
| Model 3 | **1.00 (Reference)** | | **1.59 (0.55– 4.54)** | **2.36 (0.88– 6.36)** | **2.95 (1.14– 7.62)** | **1.03(1.01– 1.05)** | **< 0.001** |  |
| **Male** | 2011 | | 1575 | 1463 | 1356 |  |  |  |
| Case number, n (%) | 43 (2.14) | | 73 (4.63) | 76 (5.19) | 81(5.97) |  |  |  |
| Incidence rate per 1,000 | 15.37 | | 35.07 | 40.56 | 46.66 |  |  |  |
| Model 3 | **1.00 (Reference)** | | **2.14 (1.47– 3.13)** | **2.42 (1.66– 3.53)** | **2.79 (1.90– 4.08)** | **1.03 (1.02– 1.05)** | **< 0.001** |  |
| **Age, year** |  | |  |  |  |  |  | 0.319 |
| **< 40** | 809 | | 704 | 690 | 787 |  |  |  |
| Case number, n (%) | 11 (1.36) | | 21 (2.98) | 35 (5.07) | 50 (6.35) |  |  |  |
| Incidence rate per 1,000 | 9.05 | | 21.53 | 36.90 | 47.34 |  |  |  |
| Model 3 | **1.00 (Reference)** | | **2.30 (1.11– 4.80)** | **3.52 (1.78– 6.98)** | **4.10 (2.08– 8.10)** | **1.05 (1.03– 1.07)** | **< 0.001** |  |
| **≥ 40** | 1354 | | 1060 | 1026 | 1022 |  |  |  |
| Case number, n (%) | 75 (5.95) | | 59 (6.68) | 57 (7.03) | 68 (7.72) |  |  |  |
| Incidence rate per 1,000 | 20.81 | | 46.73 | 48.92 | 61.06 |  |  |  |
| Model 3 | **1.00 (Reference)** | | **2.01 (1.33– 3.02)** | **2.18 (1.44– 3.30)** | **2.52 (1.67– 3.80)** | **1.03 (1.01– 1.04)** | **< 0.001** |  |

Model 1 was adjusted for age (years), sex. Model 2 was adjusted for model 1 plus education level (college and above or not) and drinking status (none, mild, moderate). Model 3 was adjusted for model 2 plus waist circumference, eGFR, ALT and antidiabetic, lipid–lowering, or antihypertensive medications usage at exam1.

^*^ Test for trend based on variable containing median value for each quarter.

**Supplementary Table 11. Previous studies of the relationship between Life’s Simple 7 score and risk of NAFLD transitions.**

| **Study (Author, Year)** | **Journal, publication date** | **Name of Cohort(s)** | **Location** | **Baseline Year** | **Participants** | **CVH metrics** | **Outcomes** | **Follow– up**  **(Years)** |
| --- | --- | --- | --- | --- | --- | --- | --- | --- |
| Oni E[10], 2000 | Am J Med, 2021 | the Multi– Ethnic Study of Atherosclerosis (MESA) | United States | 2000– 2002 | 6,814 | Life’s Simple 7 | risk of NAFLD | 0 |
| Jang EH[11],2011 | J Clin Med, 2019 | the Kangbuk Samsung Health Study | Korea | 2011– 2016 | 37,517 NAFLD cohort/93,500 NAFLD free cohort | Life’s  Simple 7 | Development  and regression of NAFLD | 5 |
| Shim SY[12] | Clin Hypertens 2023 | the Cardiovascular and Metabolic  Diseases Etiology Research Center (CMERC) cohort  study | Korea | 2013– 2018 | 2,928 | Life’s Simple 7 | risk of NAFLD | 0 |
| Liu HM[13] | Liver Int, 2019 | the Jidong and Kailuan communities | China | 2010– 2014 | 10,511 | Life’s  Simple 7 | risk of NAFLD | 0 |
| Mazidi M[14] | Atherosclerosis, 2019 | the National Health and Nutrition Examination Survey (NHANES) | United States | 1999– 2010 | 23,227 | Life’s  Simple 7 | risk of NAFLD | 0 |
| Fan H[15] | Nutrients, 2022 | The National Health  and Nutrition Examination Survey (NHANES) | United States | 2017–2018 | 2,679 | Life’s  Simple 7 | risk of hepatic steatosis  and liver fibrosis. | 0 |
| DeCoste LR[16] | Hepatol Commun, 2020 | Framingham Heart Study | United States | 2002– 2005/  1998– 2001 | 2,773 | Life’s  Simple 7 | risk of NAFLD | 0 |
| Van Dongen C [17] | Hepatol Commun. 2022 | NHANES III, 1988–1994 | United States | 1988–1994 | 3236 | Life’s  Simple 7 | all‐cause mortality and liver mortality in NAFLD with sarcopenia | 22.8 |
| Wang L [18] | Am J Med  , 2018 | a community cohort | China | 2010-2015 | 3,424 | Life’s  Simple 7 | risk of NAFLD | 5 |


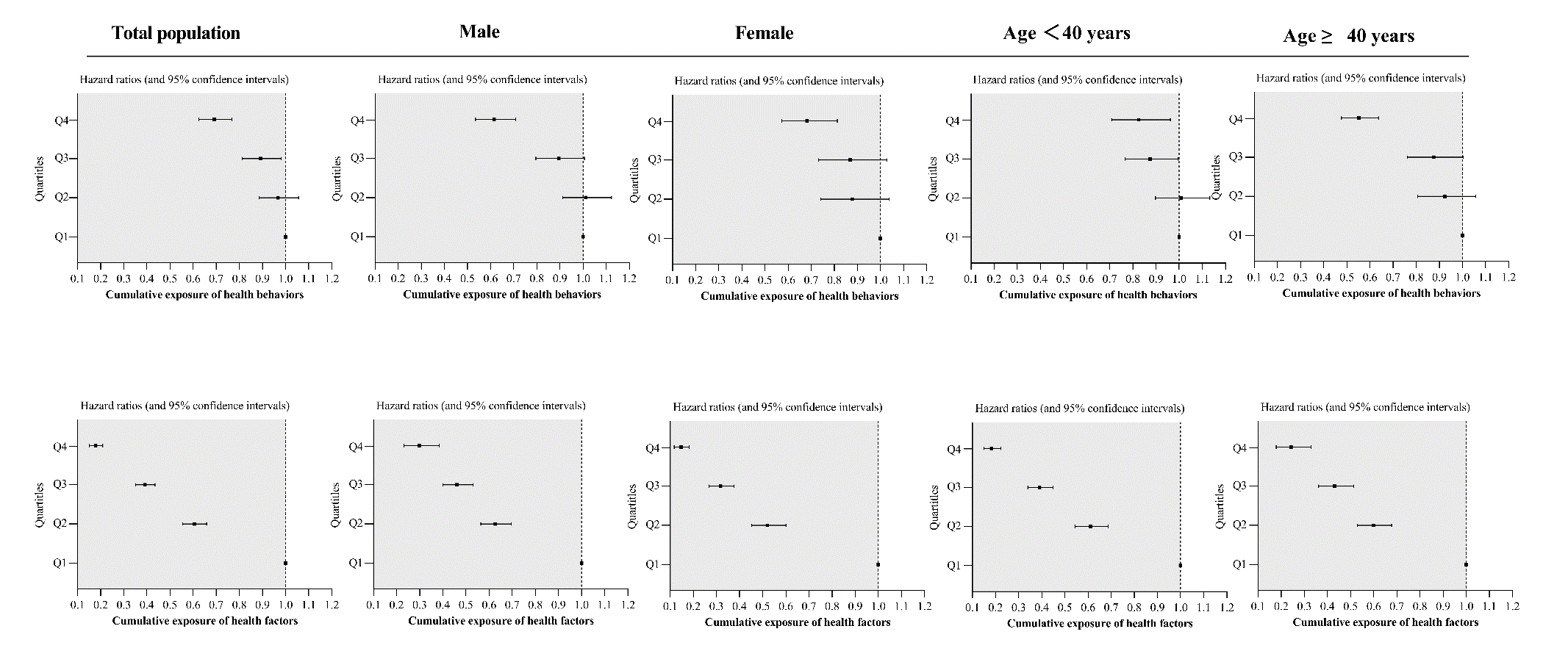
**Supplementary Figure 1.** Risks of NAFLD development in relation to quartile increase in cumulative exposure of ideal health behaviors (diet, physical activity, nicotine exposure, and sleep duration) and ideal health factors (BMI, non- HDL- C, blood glucose, and blood pressure) (n = 21,844). The models were adjusted for age, sex, education level, drinking status, waist circumference, eGFR, ALT at exam1, and antidiabetic, lipid– lowering, or antihypertensive medications usage before Exam4.


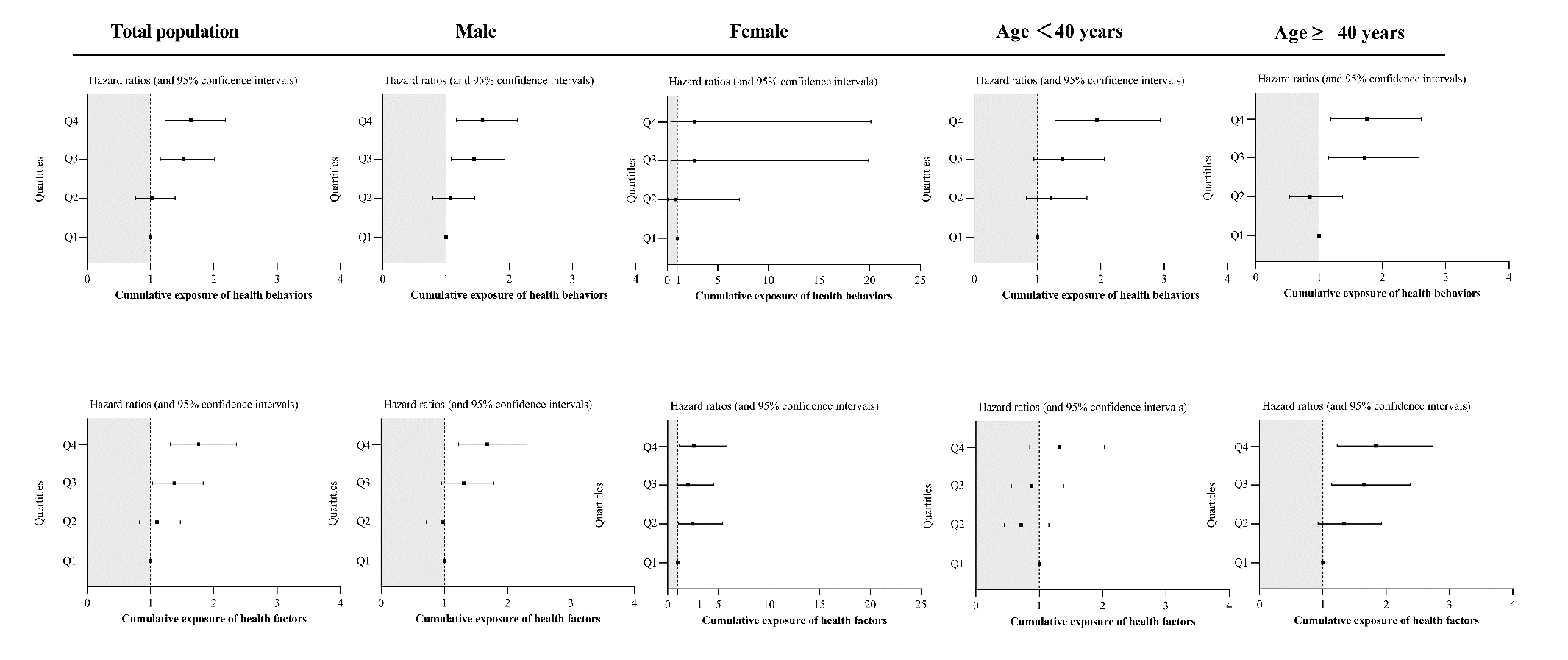
**Supplementary Figure 2.** Risks of NAFLD regression in relation to quartile increase in cumulative exposure of ideal health behaviors (diet, physical activity, nicotine exposure, and sleep duration) and ideal health factors (BMI, non-HDL- C, blood glucose, and blood pressure) (n = 6,566). The models were adjusted for age, sex, education level, drinking status, waist circumference, eGFR, ALT at exam1, and antidiabetic, lipid– lowering, or antihypertensive medications usage before Exam4.

**Reference**

[1] P.S. Bachorik, and J.W. Ross, National Cholesterol Education Program recommendations for measurement of low-density lipoprotein cholesterol: executive summary. The National Cholesterol Education Program Working Group on Lipoprotein Measurement. Clin. Chem. 41 (1995) 1414-20.

[2] J.W. Chung, S.R. Lee, E.K. Choi, S.H. Park, H. Lee, J. Choi, M. Han, H.J. Ahn, S. Kwon, S. Lee, K. Han, S. Kim, S. Oh, and G.Y.H. Lip, Cumulative Alcohol Consumption Burden and the Risk of Stroke in Young Adults: A Nationwide Population-Based Study. Neurology 100 (2023) e505-e515.

[3] P.A. James, S. Oparil, B.L. Carter, W.C. Cushman, C. Dennison-Himmelfarb, J. Handler, D.T. Lackland, M.L. LeFevre, T.D. MacKenzie, O. Ogedegbe, S.C. Smith, Jr., L.P. Svetkey, S.J. Taler, R.R. Townsend, J.T. Wright, Jr., A.S. Narva, and E. Ortiz, 2014 evidence-based guideline for the management of high blood pressure in adults: report from the panel members appointed to the Eighth Joint National Committee (JNC 8). JAMA 311 (2014) 507-20.

[4] W. Kerner, J. Bruckel, and A. German Diabetes, Definition, classification and diagnosis of diabetes mellitus. Exp. Clin. Endocrinol. Diabetes 122 (2014) 384-6.

[5] W. Yang, J. Xiao, Z. Yang, L. Ji, W. Jia, J. Weng, J. Lu, Z. Shan, J. Liu, H. Tian, Q. Ji, D. Zhu, J. Ge, L. Lin, L. Chen, X. Guo, Z. Zhao, Q. Li, Z. Zhou, G. Shan, J. He, D. China National, and I. Metabolic Disorders Study, Serum lipids and lipoproteins in Chinese men and women. Circulation 125 (2012) 2212-21.

[6] P.E. Stevens, A. Levin, and M. Kidney Disease: Improving Global Outcomes Chronic Kidney Disease Guideline Development Work Group, Evaluation and management of chronic kidney disease: synopsis of the kidney disease: improving global outcomes 2012 clinical practice guideline. Ann. Intern. Med. 158 (2013) 825-30.

[7] D.M. Lloyd-Jones, N.B. Allen, C.A.M. Anderson, T. Black, L.C. Brewer, R.E. Foraker, M.A. Grandner, H. Lavretsky, A.M. Perak, G. Sharma, W. Rosamond, and A. American Heart, Life's Essential 8: Updating and Enhancing the American Heart Association's Construct of Cardiovascular Health: A Presidential Advisory From the American Heart Association. Circulation 146 (2022) e18-e43.

[8] A. Xing, X. Tian, Y. Wang, S. Chen, Q. Xu, X. Xia, Y. Zhang, X. Zhang, A. Wang, and S. Wu, 'Life's Essential 8' cardiovascular health with premature cardiovascular disease and all-cause mortality in young adults: the Kailuan prospective cohort study. Eur J Prev Cardiol 30 (2023) 593-600.

[9] L. Li, Z. Wan, T. Geng, Q. Lu, K. Zhu, Z. Qiu, X. Zhang, Y. Liu, Q. Tian, L. Liu, A. Pan, Z. Shan, and G. Liu, Associations of healthy dietary patterns with mortality among people with prediabetes. Eur. J. Nutr. (2022).

[10] E. Oni, O. Ogunmoroti, N. Allen, A.M. MH, R. Blankstein, S.S. Martin, I. Zeb, M. Cushman, P.H. Joshi, M.J. Budoff, M.J. Blaha, R.S. Blumenthal, E. Veledar, and K. Nasir, Life's Simple 7 and Nonalcoholic Fatty Liver Disease: The Multiethnic Study of Atherosclerosis. Am. J. Med. 134 (2021) 519-525.

[11] E.H. Jang, Y. Chang, S. Ryu, S. Kim, Y.H. Kim, K.C. Sung, Y.K. Cho, S.J. Lee, H. Shin, S.H. Wild, and C.D. Byrne, Cardiovascular Health Metrics in the Development and Regression of Nonalcoholic Fatty Liver Disease: A Cohort Study. J Clin Med 8 (2019).

[12] S.Y. Shim, S.J. Jung, S.U. Kim, and H.C. Kim, Ideal cardiovascular health metrics and the risk of nonalcoholic fatty liver disease in Korean adults. Clin Hypertens 29 (2023) 3.

[13] H. Liu, Y. Yao, Y. Wang, L. Ma, X. Liu, S. Guo, X. Feng, Y. Chen, X. Chen, Z. Liu, L. Ji, D. Li, and Y. Zhou, Ideal cardiovascular health metrics and the risk of non-alcoholic fatty liver disease: A cross-sectional study in northern China. Liver Int 39 (2019) 950-955.

[14] M. Mazidi, N. Katsiki, D.P. Mikhailidis, and M. Banach, Ideal cardiovascular health associated with fatty liver: Results from a multi-ethnic survey. Atherosclerosis 284 (2019) 129-135.

[15] H. Fan, C. Xu, W. Li, Y. Huang, R. Hua, Y. Xiong, Y. Yang, X. Feng, Z. Wang, Z. Yuan, and J. Zhou, Ideal Cardiovascular Health Metrics Are Associated with Reduced Severity of Hepatic Steatosis and Liver Fibrosis Detected by Transient Elastography. Nutrients 14 (2022).

[16] L.R. DeCoste, N. Wang, J.N. Palmisano, J. Mendez, U. Hoffmann, E.J. Benjamin, and M.T. Long, Adherence to Ideal Cardiovascular Health Metrics Is Associated With Reduced Odds of Hepatic Steatosis. Hepatol Commun 5 (2021) 74-82.

[17] Van Dongen C, Paik JM, Harring M, Younossi Y, Price JK, Kabbara K, Golabi P, Younossi ZM. Sarcopenia, healthy living, and mortality in patients with chronic liver diseases. Hepatol Commun. 2022 Nov;6(11):3140-3153.

[18] L. Wang, M. Li, Z. Zhao, M. Xu, J. Lu, T. Wang, Y. Chen, S. Wang, M. Dai, Y. Hou, X. Wu, L. Ma, L. Li, S. Liu, W. Wang, Y. Xu, Y. Bi, and G. Ning, Ideal Cardiovascular Health Is Inversely Associated with Nonalcoholic Fatty Liver Disease: A Prospective Analysis. Am. J. Med. 131 (2018) 1515 e1-1515 e10.
